# On Results during 1917 and 1918

**Published:** 1919-02-22

**Authors:** 


### WORK OF INSPECTORS OF FOODS.

### On Results During 1917 and 1918.

Dr. MacFadden's report on the work of Inspectors of Foods for the year 1917-18 contains items of interest. Dr W. G. Savage has discovered a new bacillus in cases of poisoning by chitterlings, which seem to be some part or parts of a pig. Whether any other animal possesses a chitterling, or an organ homologous with a chitterling, we do not know. It sounds as if it were an innocent, harmless kind of joint or giblet, but it belies its appearance, for it, or they, was, or were, responsible for the illness of seventy-seven persons, and the deaths of four. The cases all occurred in the Provinces, and, as far as we know, chitterlings are not a Metropolitan delicacy, at least they have never been offered to us at the Carlton or the Ritz.

Liquid eggs form the subject of a long and interesting essay. We learn that a dozen liquid eggs, contained in one tin or bottle, may weigh as little as twelve ounces, which is about half the weight of twelve lawful English eggs. But those of us who have lived in the Far East—and liquid eggs are mainly a Chinese product—know that in Far-Eastern countries the eggs are very small, a peculiarity that may be associated with the practise of Far-Eastern people of allowing their fowls to get their living as they can, without the assistance of even damaged grain at considerably higher prices than sound grain, with which our own fowls are encouraged to lay eggs, larger, it is true, than Chinese eggs preserved in liquid form, but also much more than twice as costly. Liquid eggs are preserved, it appears, by the aid of boric acid, the quantity of this preservative displaying a pleasing variety, and being sometimes so little that when the eggs are made into cakes the consumer will consume only five grains per pound of cake, while in other cases he may obtain as many as 113.4 grains for the same money. It seems a generous bargain, but those who are fond of a diet of boric acid might prefer to purchase it separately and consume it by itself.

Dr. MacFadden prefers dried eggs to liquid eggs. Dried eggs are, he says, a wholesome and useful foodstuff, and no doubt, as he says so, it is so; but we note that he does not say they are appetising, and he does not explain why they become so bitter when they are kept for a week or two. It cannot be because he does not know, and we can only suppose that he wishes to keep it a secret.

